# Safety of growth hormone (GH) treatment in GH deficient children and adults treated for cancer and non-malignant intracranial tumors—a review of research and clinical practice

**DOI:** 10.1007/s11102-021-01173-0

**Published:** 2021-07-25

**Authors:** Margaret C. S. Boguszewski, Adriane A. Cardoso-Demartini, Cesar Luiz Boguszewski, Wassim Chemaitilly, Claire E. Higham, Gudmundur Johannsson, Kevin C. J. Yuen

**Affiliations:** 1 Departamento de Pediatria, Universidade Federal do Paraná, Avenida Agostinho Leão Junior, 285 - Alto da Glória, Curitiba, PR 80030-110 Brazil; 2 Departamento de Pediatria, Hospital de Clínicas da Universidade Federal do Paraná, Curitiba, Brazil; 3 SEMPR, Serviço de Endocrinologia e Metabologia, Departamento de Clínica Médica, Hospital de Clínicas da Universidade Federal do Paraná, Curitiba, Brazil; 4 Departments of Pediatric Medicine-Endocrinology and Epidemiology-Cancer Control, St. Jude Children’s Research Hospital, Memphis, USA; 5 Department of Endocrinology, Christie Hospital NHS Foundation Trust and University of Manchester, Manchester Academic Health Science Centre, Manchester, UK; 6 Department of Endocrinology, Sahlgrenska University Hospital, Gothenburg, Sweden; 7 Department of Internal Medicine and Clinical Nutrition, Institute of Medicine, Sahlgrenska Academy, University of Gothenburg, Gothenburg, Sweden; 8 Barrow Pituitary Center, Barrow Neurological Institute, Departments of Neuroendocrinology and Neurosurgery, University of Arizona College of Medicine and Creighton School of Medicine, Phoenix, AZ USA

**Keywords:** Growth hormone deficiency, Cancer survivor, Childhood cancer survivor, Growth hormone safety

## Abstract

Individuals surviving cancer and brain tumors may experience growth hormone (GH) deficiency as a result of tumor growth, surgical resection and/or radiotherapy involving the hypothalamic-pituitary region. Given the pro-mitogenic and anti-apoptotic properties of GH and insulin-like growth factor-I, the safety of GH replacement in this population has raised hypothetical safety concerns that have been debated for decades. Data from multicenter studies with extended follow-up have generally not found significant associations between GH replacement and cancer recurrence or mortality from cancer among childhood cancer survivors. Potential associations with secondary neoplasms, especially solid tumors, have been reported, although this risk appears to decline with longer follow-up. Data from survivors of pediatric or adult cancers who are treated with GH during adulthood are scarce, and the risk versus benefit profile of GH replacement of this population remains unclear. Studies pertaining to the safety of GH replacement in individuals treated for nonmalignant brain tumors, including craniopharyngioma and non-functioning pituitary adenoma, have generally been reassuring with regards to the risk of tumor recurrence. The present review offers a summary of the most current medical literature regarding GH treatment of patients who have survived cancer and brain tumors, with the emphasis on areas where active research is required and where consensus on clinical practice is lacking.

## Introduction

The large-scale production of biosynthetic growth hormone (GH) since 1985 has allowed the widespread treatment of children with different conditions associated with short stature and the treatment of adults with growth hormone deficiency (GHD), with approved indications varying among countries [1]. Some cancer survivors may require treatment with GH due to the development of GHD related to the malignancy and/or to adverse effects of its treatment, including chemotherapy, surgery, radiotherapy and biological therapy (antigen–specific monoclonal antibodies and cytotoxic T-cells) [2]. The treatment of malignancies has greatly improved over the past four decades, with a substantial increase in the number of cancer survivors. Poor longitudinal growth and GHD are often a consequence of cancer treatment during childhood [3]. In adult life, GH replacement therapy attenuates the clinical features and comorbities associated with GHD. This review presents a summary of the most current medical literature related to safety of GH treatment in children and adults who have survived cancer and brain tumors, with emphasis on the main questions where consensus on clinical practice is lacking (Table 1).

**Table 1:** Main open questions related to safety of growth hormone (GH) treatment in GH deficient children and adults treated for cancer and non-malignant intracranial tumors

Open questions
How to translate data from experimental and epidemiological studies to clinical practice?
Is GH therapy associated with a higher risk of recurrence of the primary cancer/tumor or development of a secondary neoplasia?
Is there any evidence that treatment with GH can increase the risk of death from cancer?
Which patients previously treated for cancer should be considered for GH therapy?
Should GH therapy be considered in patients with cancer-predisposing syndromes or strong family history of cancer?
What is the optimal interval between completing cancer therapy and starting GH therapy?
Are there any specific side effects that may occur after short- and long-term GH therapy?
Should pituitary tumor remnant after primary surgery be monitored and treated differently in those receiving long-term GH therapy?

### Cancer incidence

Approximately 360,000 documented cases of cancer occurred in children in 2015 [4], and it is a major cause of death worldwide. The cancer incidence rate among 0–19-year-old individuals has been reported at 155,8 per million person-years with numbers slightly higher in boys than in girls [5]. The most common cancers in childhood are leukemias (28.8%), central nervous system (CNS) tumors (24.0%) and lymphomas (11.2%) [4]. Acute lymphoblastic leukemia (ALL) accounts for approximately 80% of leukemia cases in childhood, with event-free survival rates approaching 90% with advanced multiagent chemotherapy [6]. Medulloblastoma, an embryonal tumor of the posterior fossa, is the most frequent malignant CNS neoplasm in children, and is most often diagnosed before 15 years of age. Craniopharyngioma accounts for about 5–10% of pediatric CNS tumors [7–9]. Lymphomas are the third most frequent childhood malignancy [4, 5, 10], including Hodgkin and non-Hodgkin lymphomas, with considerable risk for secondary malignancies [10].

### Growth hormone treatment and cancer in different cohorts of patients

A possible association between GH treatment and malignancy emerged from cases of leukemia in GH treated patients reported in the 1980s. Until de year 1992, 31 cases were reported, including recipients of pituitary derived GH and patients with known risk factors for malignancy [11, 12]. This finding has led to the evaluation of malignancy risk in GH-treated subjects [13–18], especially in childhood cancer survivors (CCS) with GHD [16–29]. Table 2 shows studies addressing this issue including patients treated with GH due to different indications. Population-based cohorts, long-term surveillance studies from pharmaceutical companies and collaborative international cohort studies, including SAGhE (Safety and Appropriateness of Growth Hormone Treatments in Europe, with 396,344 person-years, averaging 16.5 years per patient), showed that the overall risk of primary cancer was not increased in patients who had received GH treatment and who did not have previous risk factors for malignancy. These cohorts included children with isolated GHD, idiopathic short stature and being born small for gestational age [17, 18, 28–30]. In contrast, an increased risk of malignancy was suggested in those children who received GH therapy and had underlying conditions that are associated with an increased predisposition to cancer, including RASopathies such as Noonan syndrome, chromosomal breakage syndromes or DNA-repair disorders, such as Fanconi’s anemia and Bloom syndrome [30, 31]. An increased risk for malignancy was also found in patients treated with CNS radiotherapy in some [18, 26, 27, 32, 33], but not all studies [2, 19–21, 25, 26]. Studies in patients with benign intracranial tumors, including craniopharyngioma, did not show an increased risk of recurrence of these tumors in those individuals treated with GH [25, 26, 34–36].

**Table 2:** Studies addressing malignancy risk and cancer recurrence in patients treated with growth hormone

References	Study groups	New malignancy / Recurrence	Authors conclusions
Arslanian et al., 1985 [19]	34 CNS tumors: germinomas (4), craniopharyngiomas (18), astrocytomas (3), medulloblastomas (2), others (7); 94% GHD	GH-treated (24/34): 8 (33%) recurrences. Follow-up 8–72 months after GH therapy initiation; Non-treated (10/34): 3 (30%) recurrences	GH therapy probably is not associated with tumor recurrence
Clayton et al., 1987 [20]	Medulloblastoma (14), ALL (6), gliomas (8), ependymomas (2), leukemia (6), lymphoma (1); all GHD	21 GH-treated: 5 (23,8%) recurrences (1 optic nerve glioma, 2 medulloblastomas, 2 ependymomas); 3 during and 2 after GH treatment	No increased risk of relapse of medulloblastoma, glioma, and leukemia
Corrias et al., 1997 [21]	GHD patients irradiated for brain tumors: 25 GH-treated (11 medulloblastomas, 8 gliomas, 6 ependymomas); Control group: 100 non-GH-treated	GH-treated: 4 tumor recurrences (16%); Control group: 18 tumor recurrences (18%)	No increased risk of brain tumor recurrence after radiotherapy and GH therapy
Nishi et al., 1999 [13]	Japanese cohort of 32,000 GHD patients on treatment from 1975 to 1997	14 cases of leukemia and 1 myelodysplastic syndrome (6 with risk factors). 9 cases observed in patients without risk factors vs. 6.96–9.28 expected	Incidence of leukemia in GH-treated patients without risk factors is not increased
Leung et al., 2002 [22]	43 CCS after ALL, GHD, GH treatment for 1 to 8 years. Control group: 544 CCS after ALL, non-GH treated	GH-treated: no leukemia relapse, 1 sclerosing sweat duct carcinoma of the scalp, 1 myelodysplastic syndrome; Control group: 8 leukemia relapses, 16 second tumors	GH replacement is safe in CCS after ALL with GHD
Sklar et al., 2002 [23]	CCS: 361 treated with GH for 4.6 years (0.1–14), and 12,963 non-GH-treated. Follow-up: 6.2 years (0.4–20.6)	GH-treated: 9 recurrences; 16 second tumors (15 solid tumors, none leukemia); Control group: 502 recurrences, 344 second tumor; RR of second neoplasia: 3.21 (95% CI = 1.88–5.46; *P* < 0.0001)	No increased risk of cancer recurrence or death in CCS treated with GH, but increased risk of a secondary solid tumor
Ergun-Longmire et al., 2006 [24]	CCS patients: 361 GH-treated for 4.6 years (0.1–14); 13,747 non-GH-treated. Follow-up: 5 years	GH-treated: 20 second tumors (9 meningiomas); Non-GH-treated: 555 second tumors (62 meningiomas) (RR = 2.15; 95% CI = 1.3–3.5; *P* < 0.002)	CCS GH-treated have increased risk of second solid tumor, but risk appears to decrease with increasing length of follow-up
Wilton et al., 2010 [14]	KIGS (Pfizer International Growth Database): 58,603 patients (54% IGHD, 11% TS, 7% SGA, others). Mean follow-up: 3.6 years (197 173 patient-years)	32 new malignancies (9 CNS tumors, 3 NHL, 3 leukemias, 3 testicular cancers, 14 others); All cohort: SIR 1.26 (95% CI 0.86–1.78); TS: SIR 2.21 (95% CI 0.89–4.56); SGA: 1.61 (95% CI 0.18–5.82); Craniopharyngioma: SIR 3.24 (95% CI 0.36–11.7)	The incidence of cancer in young GH-treated patients without known risk factors for cancer is similar to the incidence in the general population
Mackenzie et al., 2011 [25]	Brain-irradiated CCS: 110 GH-treated for 8 years (4–10), follow-up of 14.5 years (11–22); 110 non-GH-treated, follow-up of 15 years (10–20)	GH treated: 6 tumor recurrence, 5 second tumors (4 meningiomas); Non-GH treated: 8 tumor recurrence, 3 second tumors (2 meningiomas)	No increased risk for recurrence or second neoplasm
Patterson et al. 2014 [26]	Childhood Cancer Survivor Study: 12,098 CCS, 338 GH-treated, 11,760 non-GH-treated. Follow-up 15 years	GH-treated: 16 (4.7%) second tumors (10 meningiomas, 6 gliomas), 8 (2.4%) recurrences. RR = 1.6 (95% CI 0.5–4.9, *P* = 0.39); Non-GH-treated: 203 (1.7%) CNS tumors (49 gliomas, 138 meningiomas, 16 others), 178 (1.5%) recurrences	No increased risk of CNS second tumor after GH-therapy. Meningiomas are more frequent. Increased risk of glioma after high dose of cranial radiation
Brignardello et al., 2015 [27]	49 GHD CCS patients (34 brain tumors, 10 ALL, 5 AML), 45 with cranial irradiation. 26 GH-treated; 23 non-GH-treated. Median follow-up: 16 years	GH-treated: 8 second tumors (5 meningiomas); Non-GH-treated: 6 second tumors (4 meningiomas). None second tumor in 4 patients with GHD without radiotherapy	No increased risk of secondary tumor on GH-treated CCS. Radiotherapy is the most important risk factor for development of second tumor
Libruder et al., 2016 [15]	GH-treated patients: 1687 low-risk (IGHD, SGA; follow-up 6.5 ± 4.0 years) and 440 intermediary-risk (MPHD, TS, SPW; follow-up 8.1 ± 4.6 years)	Low-risk group: 2 cases of malignancy, SIR 0.76 (95% CI 0.09–2.73); Intermediary-risk group: 4 cases of malignancy, SIR 4.52 (95% CI 1.22–11.57)	No increased risk of cancer in the low-risk group. Increased risk in the intermediary-risk group
Swerdlow et al., 2017 [17]	SAGhE (Safety and Appropriateness of Growth Hormone Treatments in Europe): 23,984 GH-treated patients: 52% isolated growth failure (GHD, ISS, SGA), 14.6% TS, 10.4% MPHD, 9.3% CNS tumor, others. Mean follow-up: 14.8 years for cancer incidence	SIR 2.8 for bone (95% CI 1.1–7.5) and 16.3 for bladder (5.2–50.4) in GH-treated patients without previous cancer. Cancer risk not related to GH dose, but risk of cancer mortality increased with increasing daily dose for patients treated after previous cancer	No raised risk in patients with isolated growth failure. Increased incidence of bone and bladder cancer in non-CCS group. Increased cancer mortality risk with increasing daily GH-dose in CCS
Swerdlow et al., 2019 [18]	SAGhE (Safety and Appropriateness of Growth Hormone Treatments in Europe): 10,403 GH-treated patients: 38% isolated growth failure (GHD, ISS, SGA), 16.5% TS, 12.9% organic MPHD, 12.6% CNS tumor, others. Mean follow-up: 14.9 years (154,795 person-years)	Non-cancer group: 1 meningioma (TS), SIR: 2.4 (95% CI 0.3–16.7); CCS (n = 1830): 37 meningiomas, SIR 466.3 (95% CI 337.8–643.5). CCS with previous radiotherapy (n = 1178): 30 meningiomas, SIR 658.4 (95% CI 460.4–941.7)	Risk of meningiomas: > 300-fold for CCS; > 600-fold for CCS treated with cranial (-spinal) radiotherapy. Not related to mean daily or cumulative dose and duration of GH therapy
Child et al., 2019 [29]	GeNeSIS (Genetics and Neuroendocrinology of Short Stature International Study): 22,311 GH-treated children: 58% GHD, 4.8% GHD after CNS tumor, 13% ISS, 8% TS, 6% SGA; 20,556 no previous cancer, 622 previous cancer. Follow-up 4.2 ± 3.2 years (92,000 person-year); 456 non-GH treated (192 no previous cancer, 114 previous cancer)	14 primary cancers in patients without cancer history (mainly lymphomas), SIR 0.71 (95% CI 0.39–1.20); 31 second tumors in CCS [5.0%; 10.7 (7.5, 15.2) cases/1000 PY]); CNS tumor survivors: 67 (8.1%) intracranial recurrence (SIR 16.9 (95% CI 13.3–21.47); Untreated group (114 with previous cancer): 9 recurrences, 10 second tumors	No increased risk for primary cancers

*ALL* acute lymphoblastic leukemia, *AML* acute myeloid leukemia, *CCS* childhood cancer survivors, *CI* confidence interval, *CNS* central nervous system, *GH* growth hormone, *GHD* growth hormone deficiency, *HL* Hodgkin’s lymphoma, *IGHD* idiopathic growth hormone deficiency, *ISS* idiopathic short stature, *MPHD* multiple pituitary hormone deficiency, *NHL* non-Hodgkin’s lymphoma, *PWS* Prader-Willi syndrome, *RR* relative risk, *SGA* small for gestational age, *SIR* standardized incidence ratio, *SMR* standardized mortality, *TS* Turner syndrome

## GH/IGF-I and cancer – background

### Experimental evidence for the role of GH-IGF-I system in carcinogenesis

The effects of GH in stimulating mitosis, cell differentiation and growth has been known since the early years of the twentieth century [37, 38]. In the 1950s, it was shown that GH action in peripheral tissues could be mediated by insulin-like growth factor I (IGF-I) [39], and since then, novel components of the GH-IGF-I signaling system and their roles on normal and abnormal cell growth and metabolism have been progressively unraveled [37–39].

**Fig. 1:**
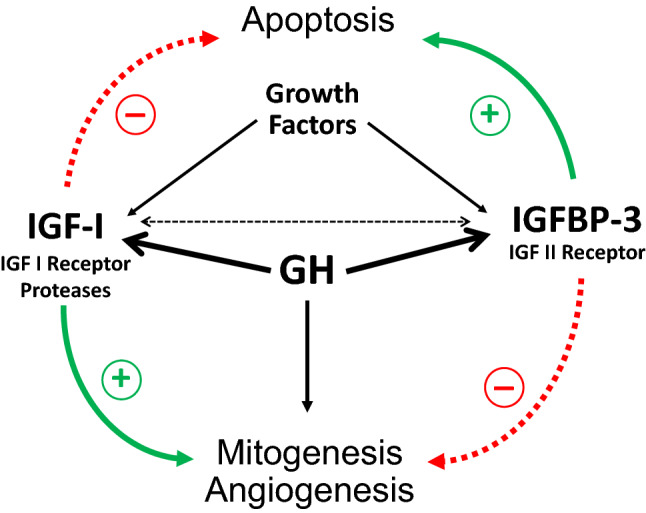
Diagrammatic representation of endocrine and paracrine effects of GH on mitogenesis, angiogenesis and apoptosis, either directly or by synergy with other growth factors. GH stimulates both IGF-I and IGBP3 [41]. While IGF-I and IGFBP proteases favors cell proliferation and inhibits apoptosis [43, 44, 47], IGFBP3 [46] and IGF-II receptors [47] act in the opposite direction, via an IGF-independent pathway. The result of these opposed forces in a tissue-specific environment might be critical for normal and abnormal cell growth [41]. Dotted lines: inhibition. Continuous line: stimulation. Adapted from Ref. [46, 48]

The mechanisms involved in the control of cell growth, differentiation and death are tightly regulated by a complex cascade of molecular events that, when disrupted, can lead to an increased risk of malignant transformation. Experimental models have shown the ability of endocrine and paracrine GH and IGF-I to promote cell proliferation and differentiation, angiogenesis, and inhibition of apoptosis, either directly or by synergy with other growth factors [30, 40–44]. In the multistep process of tumorigenesis, GH and IGF may potentially increase the number of mutations by reducing time for DNA repair during rapid progression of neoplastic cells [45]. In contrast, other players of the GH-IGF system, such as insulin-like growth factor binding protein 3 (IGFBP3) [46] and IGF-II receptors [47], inhibit mitogenesis, stimulate apoptosis and modulate IGF-I actions, thus acting as protective factors against tumor progression [30, 40–42, 46, 48] (Fig. 1). Additionally, various solid and hematologic malignancies have been associated with local production of GH, IGF-I, and IGFBPs, normal or altered expression of several receptors of the GH-IGF system, and deregulation of miRNAs induced by GH and IGF-I [30]. The final effect of these opposed endocrine and paracrine forces of the GH-IGF system in a tissue-specific environment might be critical for normal and abnormal cell growth, but how and when GH per se may participate in this process is largely unknown.

The potential role of the GH-IGF-I system in the development of specific types of cancer has been comprehensively reviewed in several recent publications [30, 41]. It is important to note that GH-induced intracellular signaling pathways have been identified as the third most highly associated with breast cancer susceptibility among 421 pathways containing 3962 genes in a human genome-wide association study [49]. In breast cancer, pituitary and exogenous GH seems to be less involved, while local expression of GH has been shown to have profound autocrine/paracrine effects in breast tissue independent of IGF-I, leading to increased epithelial cell proliferation, and conferring an invasive phenotype on mammary carcinoma cells by affecting epithelial–mesenchymal transition [50, 51]. Moreover, a model to explain neoplastic colon growth has been proposed in which high endocrine or autocrine GH levels – for instance, as a result of acromegaly or colonic DNA damage and inflammation – inactivate tumor-suppressor genes, suppress apoptosis, and stimulates epithelial to mesenchymal transition, leading to changes in the intestinal mucosal field that favor malignant transformation [52]. Consequently, several components of the GH-IGF-I signaling cascades have been investigated as targets for the treatment of breast and colonic cancer, as well as for several other malignancies [53–56].

Cumulative data obtained from natural or genetically modified animals exhibiting normal or disrupted GH production or action, have also brought a wealth of evidence linking GH axis and carcinogenesis [56, 57]. The repression of the GH-IGF signaling system in many of these models has been associated with significant reductions in cancer rates and increased longevity. In contrast, transgenic mice with excessive circulating levels of GH or tissue overexpression of IGF-I exhibit an increased risk for hyperplasia and tumor formation [30, 58].

### Epidemiological and clinical evidence in humans

From the 1990s onwards, a substantial amount of epidemiological data has found associations between serum IGF-I levels in the highest quartiles of the normal reference range with an increased incidence of several cancers in the general population [59–63]. Moreover, associations between normal-low IGFBP3 levels and prostate cancer and breast cancer in postmenopausal women were also observed, but this association was likely influenced by the concomitant association of normal-high IGF-I levels and the presence of these tumors in the general population [60]. In the last decade, data obtained from the European Prospective Investigation into Cancer and Nutrition (EPIC cohort) involving more than 500,000 healthy volunteers have confirmed the association between higher circulating IGF-I levels and the risk of breast cancer in receptor-positive tumors in women older than 50 years, thyroid cancer, low-grade gliomas and acoustic neuromas [64–66]. Nevertheless, there are many caveats and confounding factors in these epidemiological studies, and the associations are usually modest and without a threshold IGF-I level that would allow its use in cancer screening or monitoring. Therefore, a potentially causal relationship between elevated IGF-I and a higher risk for cancer lacks the certainty needed to apply these findings in routine clinical care.

In humans, congenital GHD or GH resistance (Laron dwarfism) have been associated with a lower incidence of malignancies, similarly to what is observed in animal models [67–69]. Cells treated with serum obtained from Ecuadorian patients with Laron syndrome have been shown to exhibit reduced DNA breaks and increased apoptosis [68]. In the Ecuadorian cohort, not a single case of cancer was noted during a 22 years follow-up, while in cohorts of GHD patients due to GHRH-R defect or congenital isolated or multiple GHD, in which small amounts of circulating GH can be demonstrated, few cases of cancer have been reported both in naïve and in patients treated with GH [67]. These data suggest that the protection against cancer in human GHD is not absolute and that GHR signaling via pituitary or exogenous GH might be mitogenic in the long term. In contrast, acquired hypopituitarism has been linked to either an increased risk of having cancer or an increased risk of dying from cancer in some, but not all, retrospective studies [70, 71]. Of note, in acquired hypopituitarism, factors other than GHD may play a role in these observed associations, such as the underlying pituitary disease, inherent elevated risk for tumors in patients with pituitary adenomas, radiotherapy, associated morbidities and inadequate or inappropriate pituitary hormone replacement [70, 71]. Studies investigating the risk of cancer in acromegaly patients who are chronically exposed to very high levels of GH-IGF-I for many years have produced inconsistent and controversial findings. Acromegaly is characterized by a prolonged and excessive secretion of GH which, in turn, induces both IGF-I and IGFBP3 production, resulting in a dysregulated, unpredictable balance of cell cycle regulation, characterized by signals for cell growth competing with signals for cell death [72]. The ultimate consequences of these antagonistic mechanisms are the basis for concerns and disputes on the cancer risks in patients with active acromegaly. More recent data suggest that other factors, such as age, comorbidities, inhered predisposition and regional differences of cancer prevalence are likely to contribute equally or more to the risk of cancer in acromegaly than the GH-IGF-I levels per se [30, 72].

## GH treatment in cancer survivors in childhood

Many factors lead to reductions in longitudinal growth in CCS, including poor nutrition, low body mass index, long-term treatment with glucocorticoids, growth plate damage, background syndromic short stature and GHD. Tumors and surgeries that involve the hypothalamic-pituitary region are major risk factors for hypothalamic-pituitary axis (HPA) dysfunction, which often are present at baseline or soon after the procedure. On the other hand, CNS radiation frequently causes hypothalamic-pituitary dysfunctions several months or years after treatment [73]. GHD is the most frequent hypothalamic-pituitary disorder in CCS, with a prevalence of 22.2% in all groups and of 40.2% following radiation of hypothalamic-pituitary region [3, 74]. The average time between completion of tumor therapy and the onset of poor longitudinal growth has been reported as 37.7 months in children treated with radiotherapy [75], ranging from 3 months to 5 years [76]. The risk of short adult height is higher when radiotherapy is given at younger age and before puberty [73, 77]. GHD has been associated with radiotherapy doses ≥ 18 Gy to the hypothalamic-pituitary region area as well as with total body irradiation using a 10 Gy single dose or fractionated doses totaling 12 Gy [77]. In a study of 192 children with primary brain tumors, the authors predicted the occurrence of GHD on the basis of dose and time after irradiation: GHD appeared 12 months if the radiotherapy dose was greater than 60 Gy, in 36 months if the dose was between 25 and 30 Gy, and 60 months if the dose was between 15 to 20 Gy [78]. Morphological changes affecting the pituitary are associated with linear growth decline among patients surviving ALL [79] and nasopharyngeal carcinoma [80]. The pituitary height was significantly reduced after radiotherapy, possibly due to radiation-induced pituitary cell apoptosis, or because radiation may have affected vascular function and oxygen supply to the pituitary gland [80]. Proton based radiotherapy is increasingly used for the treatment of brain tumors, a development that carries a promise in reducing scatter to normal tissue, including the hypothalamic-pituitary area when lesions are located elsewhere in the head [81, 82].

Spinal irradiation is an independent risk factor for growth impairment; it may cause disproportionate height due to reduced growth of vertebral bodies and spinal deformities [83].The resulting skeletal dysplasia can be detected by measuring an increased sitting height to standing height ratio [84, 85]. CCS treated with higher doses of spinal radiation (> 20 Gy) at a younger age, and to a larger volume of the spine, are at increased risk of short adult height [77]. The disproportionate growth may be evident as early as one year following radiotherapy. It becomes progressively more evident during puberty [84] and GH replacement in GHD cancer survivors who also received spinal irradiation may result in an improvement in leg length but not in spinal length and total height [77]. GHD is unlikely after traditional chemotherapy, but some newer agents may interfere with normal growth by affecting growth plates or dysregulating GH-IGF-I signaling pathways (tyrosine kinase inhibitors) or causing an autoimmune hypophysitis (immune checkpoint inhibitors) [73]. Some alkylating agents, such as busulfan and lomustine, may increase the risk for short stature, particularly after leukemia or neuroblastoma, possibly by increasing the vulnerability of the hypothalamus-pituitary to damage from radiotherapy or by directly affecting the hypothalamic-pituitary axis [85]. They are also risk factors for gonadal dysfunction and hypothyroidism, which may further exacerbate growth impairment [85]. Other chemotherapy agents may directly damage the growth plates and cause severe short stature; these include cis-retinoic acid for the treatment of neuroblastoma and hedgehog pathway inhibitors [86, 87].

The growth of children who have undergone cancer treatment should be assessed every 6–12 months, and lifelong assessment may be necessary to screen for the development of GHD. It is recommended to measure standing and sitting height in children treated with spinal radiotherapy, i.e., total body irradiation, craniospinal irradiation, as well as radiation to the chest, abdomen, or pelvis [77]. GHD should be suspected when growth deceleration is observed with a deflection of at least 0.3 SDS/year or height deviates from the familial background [88]. The measurement of serum IGF-I levels is not recommended as a biomarker for the diagnosis of GHD in CCS, especially among those exposed to hypothalamic-pituitary irradiation [77]. Normal serum IGF-I levels have been observed in CCS who failed in GH dynamic testing, especially those treated with low doses of radiotherapy. In survivors of childhood ALL treated with cranial radiotherapy, IGF-I levels < −2 SDS showed a sensitivity of only 17.86%; sensitivity of IGF-I as a diagnostic tool was even lower (7.14%) in subjects treated with 14.4 Gy total body irradiation before bone marrow transplantation. The use of IGF-I as a screening tool should be restricted to patients treated with high cranial radiation doses [89]. Similarly, serum IGFBP3 do not aid in the diagnosis of GHD in CCS children [89–91].

In patients with a high probability of GHD (poor growth, other pituitary hormones deficiencies), one GH provocative test is sufficient to make the diagnosis, after excluding other growth failure etiologies (e.g., total body irradiation, imatinib mesylate and cis-retinoic acid treatment) [90]. The insulin tolerance test has been considered as the gold standard provocative stimulation test to make the diagnosis of GHD, but caution should be exerted and alternative secretagogues should be considered in patients at risk for seizures [90]. Growth hormone-releasing hormone (GHRH) alone or in combination with arginine is not recommended in CCS after hypothalamic-pituitary irradiation because radiation doses less than 40 Gy cause predominantly hypothalamic damage, and consequently, exogenous hypothalamic hormones administration may cause false-negative results [74, 77]. The Pediatric Endocrine Society guidelines suggest that one can make the diagnosis of GHD without using a GH dynamic test in patients with the following conditions: auxological criteria as mentioned before, hypothalamic-pituitary defect (such as major congenital malformation, tumor or irradiation), and deficiency of at least one additional pituitary hormone [92]. Provocative GH stimulation tests are also not needed in patients who have three other confirmed hypothalamic-pituitary hormone deficiencies [77]. According to Cattoni et al. [89], the peak GH level attained on a stimulation test decreases on average 0.1 μg/L for each additional Gy of pituitary exposure to radiation. It is important to avoid false-positive tests in the diagnosis of GHD, with special attention to obesity and considerations about GH assays and cutoff values [90]. Brain imaging should be performed prior to starting GH therapy to rule out a preexisting tumor and to serve as a baseline study in children whose cancer monitoring does not routinely include CNS imaging [25].

Treatment with GH is usually indicated in CCS with proven GHD after careful discussion with the patient, his/her family if appropriate, as well as with the treating oncologist or neurosurgeon. The most appropriate and safest time to start GH is still a topic of debate, primarily due to a paucity of data and lack of controlled trials [93]. The Endocrine Society and the Pediatric Endocrine Society guidelines suggest waiting for 12 months after completing cancer treatments [77, 92]. In children with residual tumor and stable disease, as is often the case with optic pathway tumors and low-grade gliomas, the safety and timing of the initiation of GH treatment should specifically be discussed with the oncologist [77]. In children with craniopharyngiomas, which are considered benign tumors, GH therapy may be safely initiated as early as 0.7 year from diagnosis [77]. The safety of GH replacement for children on treatment with tyrosine kinase inhibitors has not been established and therefore is not recommended at the present time [77]. GH doses are similar to those used for the treatment of GHD in non-CCS [77, 92], with a starting dose of 0.022–0.035 mg/kg/day (0.15–0.25 mg/kg/week) and individualization of subsequent dosing [92]. Serum IGF-I levels should be measured during the treatment and kept in the normal range for sex, age, and pubertal status [77, 88, 92]. In conditions associated with increased predisposition to malignancy, e.g., Down syndrome, Fanconi anemia, Noonan syndrome, Bloom syndrome, neurofibromatosis 1, among others, the decision of whether to start GH therapy or not is a very controversial issue [92, 93]. Table 3 summarizes the main recommendations for GH treatment in CCS.

**Table 3:** Current recommendations related to clinical evaluation and growth hormone (GH) treatment in cancer suvivors during childhood

Recommendation	
Growth assessment	Every 6–12 months
Increased risk for developing GHD	Cranial irradiation: Younger age and prepubertal children ≥ 18 Gy Larger doses and earlier GHD (ex. ≥ 60 Gy, 12 months) Total body irradiation: ≥ 10 Gy (single dose) or ≥ 12 Gy (fractionated doses) Immune checkpoint inhibitors (hypophysitis)
IGF-I measurement	Not recommended for diagnosis, except as screening of adult patients treated with high cranial radiation doses
IGFBP3 measurement	Not recommended for diagnosis
GH provocative test	Usually, only one test is necessary Not necessary if 3 or more other hypothalamic-pituitary hormones deficiencies Gold standard: ITT GHRH ± arginine not recommended after cranial irradiation
CNS imaging	Prior to GH treatment initiation
Treatment	Only with proven GHD After 12 months after the end of cancer treatment Necessary discussion with family, patient and oncologist Initial dose: 0.022–0.035 mg/kg/day To maintain serum IGF-I concentration in normal range Contraindications: use of tyrosine kinase inhibitors

*GHD* growth hormone deficiency, *IGF-I* insulin-like growth factor I, *IGFBP3* insulin-like growth factor binding protein 3, *ITT* insulin tolerance test, *GHRH* growth hormone-releasing hormone

The use of GH in CCS carries additional safety concerns. Scoliosis is common following either spinal surgery and/or spinal or craniospinal irradiation [77, 94], and GH therapy may exacerbate this condition due to rapid growth [92]. Slipped capital femoral epiphysis is more frequently seen during GH treatment in GHD following intracranial neoplasms, craniopharyngioma and after bone marrow transplantation compared with patients with idiopathic GHD. GH replacement is also associated with a slightly increased risk of intracranial hypertension [92]. Some chemotherapy agents, e.g., cisplatin, alkylating agents, anthracyclines, and camptothecins, may induce insulin resistance, hyperinsulinemia, and impaired glucose control by directly influencing insulin sensitivity [95–97]. Survivors of craniopharyngioma are at risk for hypothalamic obesity and insulin resistance [73]. Hence, cranial radiotherapy may induce GHD, which may promote the development of metabolic syndrome. Cranial radiotherapy is a major risk factor for obesity, dyslipidemia, and insulin resistance in CCS [95]. A higher body mass index before hematopoietic stem cell transplantation for childhood ALL and GHD were also associated with increased risk for metabolic syndrome [97].

## GH treatment in cancer survivors during adulthood

Adults with hypopituitarism and documented GHD have distinct clinical features that have been demonstrated in many studies [98, 99]. These include overweight, abdominal obesity, reduced lean muscle mass, decreased extracellular fluid volume, raised cholesterol and triglycerides, and low bone mineral density with likely increased vertebral fracture rate [99]. Adults with hypopituitarism including GHD also describe reduced energy and well-being, possibly due to reduced muscle strength and exercise capacity. Many of these outcomes have been shown to improve with GH replacement, which has thus garnered increasing support by expert panels over the past two decades [99–102]. Excess mortality due to cardiovascular risk factors has also been reported in adults with multiple hypothalamic-pituitary deficits including GHD. However, the specific contribution of GHD to all causes of mortality in this population has yet to be established, especially in light of markedly higher mortality rates in adults with a history of cancer or craniopharyngioma when compared to those with non-functioning pituitary adenoma [103–105]. Despite the potential benefits, the perceived increased risk for tumor recurrence and the controversy surrounding a potential association with secondary neoplasia [24] may explain provider reluctance to offer at-risk adult CCS testing for and treatment of GHD. For instance, in the St. Jude Lifetime Cohort study, more than 99% of patients with GHD were not given GH replacement therapy [106].

GHD and hypopituitarism were commonly reported late effects in CCS, in particular those surviving CNS tumors, and older patients treated for childhood leukemia during the 1970s [106–109]. The proportion of individuals receiving radiation therapy for these tumors decreased during the 1990s [109]. In general, the same diagnostic approach for GHD can be used in adult cancer survivors as in patients with hypothalamic-pituitary disorders due to other causes [100], with adjustments made to testing approaches as summarized in Sect. 3, especially in relation to the GHRH and the combined GHRH-arginine stimulation tests after brain irradiation. Macimorelin, an oral ghrelin mimetic, has demonstrated a diagnostic accuracy similar to that of the ITT, is well-tolerated and safe. In adults without other pituitary hormonal deficiencies, it is good practice to perform two stimulation tests to diagnose GHD unless IGF-1 is also below the lower limit of the reference range [99, 110]. GH replacement in adult cancer survivors involves individualized dose titration that maintains serum IGF-I levels within the age-related reference range and achieves an appropriate clinical response, for example, an improvement in the AGHDA (Adult Growth Hormone Deficiency Assessment) score [100, 101, 111, 112]. Hypopituitary women treated with GH should receive estrogen replacement preferably by non-oral route, as oral estrogen reduces the serum IGF-I response to GH therapy [113]. The expected benefits of GH treatment are the same as those reported in adults with GHD due to other causes not related to cancer [2], including reduction of total body fat mass and abdominal fat mass, improvement in lipid profile, left ventricular systolic function, muscle strength, quality of life (QoL) and cognitive performance [114–119].

Hypopituitary adults treated with GH have been reported to have a similar subsequent cancer risk compared to individuals not treated with GH [120–122], whereas a meta-analysis even suggested that the cancer risk could be reduced [123], although this observation is likely due to selection bias. Data from the ongoing PATRO pharmaceutical sponsored post-marketing surveillance study of children (*n* = 136) and adults (*n* = 293) with GHD were analyzed over 10 years of real-life clinical experience. No increased risk was seen for neoplasia compared with other GH treatments [124]. Further analysis from the same database including 1293 adults, of which 637 (49.3%) were GH treatment-naïve at study entry and the majority having multiple pituitary hormone deficiency (*n* = 1128, 87.2%), demonstrated that GH treatment did not result in an increased cancer risk, although an increased risk of second new malignancies in patients with previous cancer could not be excluded [125]. Conversely, the SAGhE studies of GH therapy in young adults with childhood-onset GHD suggested an increased risk for certain cancer types or a trend towards increased risk of mortality with GH therapy [17, 126]. These divergent results could be explained by a variety of confounding factors, possibly unrelated to GHD, including differences in sampling size and study design, types of cancers, prevalence of specific cancer types in the control populations, underlying pituitary disease, surgery, radiation, associated co-morbidities, and sub-optimal pituitary hormone replacement therapies. Therefore, clinicians have to navigate challenging safety questions when considering GH replacement therapy, especially in newly diagnosed GHD adults with a previous history of cancer and those who developed cancer while previously receiving GH. Current Endocrine Society recommendations suggest starting GH therapy after 1 year of disease remission following childhood cancer treatment [77], but it remains unknown whether 1 year is sufficient or deferring beyond 1 year is safer as there are no hard data to support this recommendation. In the case of chronic or not totally eradicable oncological disease, the choice of whether to start GH or not should be tailored according to cancer type and patient comorbidities. If GH therapy is considered, the decision should be individualized after a thorough discussion with the patient and clearance from the oncologist [77, 127]. Published guidelines do not recommend treatment and the label contains a “black box” warning against using the GH in patients with active cancer. For these patients, it may be reasonable to start GH at least two years after cancer remission if the patient has expressed a keen desire to start GH replacement therapy; however, these patients should be counseled that conclusive data on the effects of GH replacement and cancer risk are still lacking [127]. The benefits of GH replacement should be carefully balanced against the possible, yet unsubstantiated, increased cancer risk. A prospective surveillance study of a large cohort of GH-treated patients using optimal dosing and untreated GHD hypopituitary patients with a history of cancer in remission is an unmet need; however, conducting such a large and long-term study will be challenging due to the high cost and adjustments needed to be made for the differences in demographics between GH-treated and untreated subjects [128].

Adherence to daily subcutaneous GH injection is often challenging. Numerous studies have shown that most children and adults are non-adherent to daily GH injections [129, 130] leading to high treatment discontinuation rates [131, 132]. Thus, there has been a push to develop long-acting GH formulations to allow reductions of injection frequency in order to improve treatment adherence [133–135]. At present, two long-acting GH formulations have been introduced to the market in Asia, several long-acting GH formulations are in the latter stages of development, and one has been approved in the United States and Europe but is not commercially available yet [133, 135]. Current evidence indicates that long-acting GH formulations are safe in non-cancer GH-deficient patients [133, 135], although it may be argued that these studies are too short of a duration to address this question. Whether they are equally safe in GH-deficient cancer survivors especially given their differences in pharmacokinetic and pharmacodynamic profiles compared to daily GH injections requires further long-term studies.

## GH treatment and second neoplasm

The risk of developing a second neoplasm is an important consideration during follow-up of cancer survivors, as CCS are at a significantly higher risk during their lifetime. The most frequently reported second neoplasms are non-melanoma skin cancer, breast cancer, meningiomas, thyroid cancer, soft tissue sarcomas, and CNS tumors, by order of frequency [136, 137]. Genetic factors and adverse effects from cancer treatment may synergize in raising the risk among survivors [106]. Cranial radiotherapy is known to increase the risk for developing meningioma [109, 137]. While a nonsignificant association was reported between GH replacement therapy and recurrence of the primary cancer, studies have reported that treatment with GH may increase the risk for secondary neoplasia in CCS [17, 22, 23, 138–141]. In the SAGhE cohort, of 10,403 patients treated with GH, 38 were diagnosed with meningioma. Thirty of them had been treated with cranio-spinal radiotherapy, given a standardized incidence ratio (SIR) for meningioma in the overall cohort of 75.4 [18]. However, the risk of meningioma was not increased in patients whose diagnosis before GH treatment was not cancer (SIR = 2.4). There were no significant associations between this risk for meningioma and age of GH start, time since starting treatment, mean daily GH dose, duration of treatment, or cumulative dose of GH [18]. Sklar and colleagues [23] reported an increased risk of second solid neoplasms after starting GH (RR, 3.21), mainly in survivors of acute leukemia/lymphoma (RR, 4.98), with no secondary leukemias. In a control-matched study with patients treated with CNS irradiation with a follow-up of 14.5 years, the incidence of recurrent or secondary tumors did not differ significantly between GH-treated and controls [25]. The risk of GH-dependent effects on secondary tumor was 10% in both GH-treated and untreated patients with a mean latency time of 22.5 years [25]. Again, meningiomas were the most frequent second tumor in the GH-treated groups [24–26]. Female sex, young age at primary cancer diagnosis, a long time period since cranial irradiation [2], CNS radiation dose ≥ 20 Gy and cumulative doses of multiple alkylating agents [142] were associated with meningioma development. These reports reinforce the need to interpret cautiously the risk of secondary tumors in GH treated patients. It is advisable to consider the relatively small number of events, high confidence intervals and low levels of significance. In addition, the risk of outcome bias due to the fact that CCS are already at increased risk of second neoplasm [18].

## GH treatment in non-malignant sellar tumors

### Craniopharyngioma

Craniopharyngioma represents about 5–10% of pediatric CNS tumors and 3% of intracranial tumors for all age groups, with peak incidences in the age categories of 5 to 9 and 40 to 44 years old. Approximately 40–87% of children will have at least one deficient hypothalamic-pituitary hormone at time of diagnosis [7–9, 143, 144]. Craniopharyngioma is primarily treated surgically, with or without adjuvant radiotherapy [7, 8, 143]. Patients who are not GHD at diagnosis will often develop GHD post resection or following focal radiotherapy [143]. Progression rates range from 71–90% after surgery alone to 21% when radiotherapy is used after partial resection [8]. The safety concerns related to GH treatment in patients with craniopharyngioma are the potential effects of GH on growth of known residual disease or recurrence of radiologically “cured” disease following surgical intervention ± radiotherapy.

Carefully conducted case control studies provide most of the information on this tumor. The majority of these studies report on a mix of childhood and adult-onset disease. In the study of Olsson et al. [145] that included all age categories, 29% of the patients in the GH treated group had residual tumor compared to 47% in the untreated group. However, there was no difference in tumor progression between the two groups up to 15 years after treatment. Other similar studies where patients with craniopharyngiomas were studied alone or included in larger cohorts of sellar tumors, have also failed to demonstrate any evidence of craniopharyngioma recurrence with GH therapy [19, 146–150]. A recent well conducted, single centre, retrospective analysis included 89 patients with adult onset craniopharyngioma with a median duration of treatment follow-up of greater than 7 years, again demonstrated no increased risk of craniopharyngioma recurrence following initial neurosurgery in those treated with GH therapy [151].

Safety data have also been derived from pharmaceutical sponsored post marketing surveillance studies in both children and adults with craniopharyngioma. The report from the KIMS (Pharmacia & Upjohn International Metabolic Database) with 1000 adult patients registered at the time of the analysis, recorded 12% with a craniopharyngioma. Only 6 patients overall were reported as having tumor recurrence on GH therapy, none of whom had a craniopharyngioma [152]. More recently, the HypoCCS study of 1058 adults with craniopharyngioma found no association of GH treatment with risk of recurrence (RR 1.32; range 0.53–3.31, p = 0.55) with a mean follow-up of 4.8yrs [121]. Other open-label post marketing surveillance registries have similarly failed to show any increased risk of craniopharyngioma recurrence with GH treatment in childhood onset craniopharyngiomas [141, 153–155]. A recent meta-analysis of 10 studies investigating GH treatment in children with craniopharyngiomas compared 3436 patients who received GH with 51 who did not. Their results suggested that recurrence rates of craniopharyngioma were reduced in children receiving GH, although this may reflect selection bias within the individual studies towards favoring GH treatment in those with less aggressive tumors [36].

Despite the reassuring clinical data, in vitro studies have demonstrated growth of craniopharyngioma cells in culture in the presence of exogenous GH [156]. GH receptors have been identified on craniopharyngiomas [157], and increased GH receptors expression may reflect tumor aggressiveness with potential prognostic implications in some patients [158]. A diagnosis of craniopharyngioma is associated with an excess mortality rate compared to the general population, with most recent analyses demonstrating a SMR of between 2–3 [159, 160]. There is also an increased risk of metabolic complications, with data from KIMS demonstrating that patients with craniopharyngioma had a ninefold increased risk for developing diabetes mellitus compared to a background Swedish population [160]. Use of GH has not been shown to increase the risk of diabetes mellitus in this population, [159] although one study showed a decline in insulin sensitivity markers over a longer duration of GH treatment [161].

### Pituitary adenomas

Non-Functioning Pituitary Adenomas (NFPAs) are the most frequent pituitary tumor leading to adult GHD. Alongside craniopharyngiomas, NFPAs make up the majority of patients represented in the largest registries of GH replacement therapy [162, 163]. The data with regard to growth or recurrence of NFPAs with GH therapy are reassuring. The most recent and largest, single centre study, of adult patients treated surgically for NFPAs demonstrated no evidence of a difference in NFPA recurrence in 74 patients treated with GH compared to 120 patients who chose not to receive GH [151]. A retrospective case-controlled study from 2009 provided 10-year tumor progression free data in over 200 patients with NFPA and GHD, again demonstrating no difference in NFPA progression between those treated or not treated with GH [164]. These reassuring data reflect a number of other carefully conducted case-controlled studies and post-marketing surveillance studies [121, 152, 165–169] with up to 14 years of follow-up analyzed.

The safety of GH in the management of GHD in patients with an original diagnosis of acromegaly (acroGHD) is less well elucidated. A randomized placebo-controlled trial of GH in 30 patients [170] showed improved QoL and body composition with no detrimental effects or safety concerns over 6 months although an earlier open label study in 20 patients had shown increased cardiovascular events in the GH treated group [171]. Retrospective analysis of the KIMS data set, comparing acroGHD and NFPA GHD patients treated with GH to a background reference population demonstrated an increased cardiovascular mortality in the acroGHD compared to background population and the NFPA group. It was not possible to determine if this was related to the use of GH or to the previous acromegaly, but caution was advised in treating this group of patients with GH. Of note, markers of glucose tolerance showed a small but significant decline over time in both the GH treated acroGHD and NFPA group [172].

Although the data are reassuring with regard to overall risk of recurrence/regrowth of the original tumor not being increased with GH therapy, the majority of craniopharyngiomas that do recur will recur within 7 years, and those non-functioning adenomas that recur will grow within 5 years, with growth becoming less common after 10 years [173]. Many of the studies on the safety of GH treatment in these patients have a shorter duration of follow-up and therefore may underestimate recurrence and regrowth rates.

## Summary

GH replacement has been shown to improve linear growth during childhood as well as to enhance body composition, bone health, quality of life and well-being parameters in adults. Many studies and several expert panels have attempted to assess whether these benefits override potential safety concerns in individuals who have experienced a malignancy or CNS benign neoplasia. Currently published data regarding survivors of childhood and adult cancer do not suggest that GH replacement increases future cancer risk, although it remains difficult to identify factors that may modulate cancer risk in older patients, individuals with increased predisposition to malignancy and those with a strong family history of cancer. Despite the growing availability of data from cohorts with long-term follow-up, consensus on clinical practice is lacking in several areas. These include whether in-vitro pro-neoplastic properties can truly provide the basis for safety concerns related to GH replacement, whether treatment with GH with increased serum concentrations of IGF-I could independently contribute to worse tumor or mortality outcomes in at-risk populations, how to manage potential safety concerns in individuals who are GHD and are predisposed to cancer and finally, whether best practices in the management of GHD could reduce some of the risk potentially conferred by GH replacement, and whether long-term use of long-acting GH preparations may exacerbate the risk of cancer in CCS.
